# Genetic Polymorphisms of *XRCC1*, Alcohol Consumption, and the Risk of Colorectal Cancer in Japan

**DOI:** 10.2188/jea.JE20110059

**Published:** 2012-01-05

**Authors:** Guang Yin, Makiko Morita, Keizo Ohnaka, Kengo Toyomura, Nobuyuki Hamajima, Tetsuya Mizoue, Takashi Ueki, Masao Tanaka, Yoshihiro Kakeji, Yoshihiko Maehara, Takeshi Okamura, Koji Ikejiri, Kitaroh Futami, Yohichi Yasunami, Takefumi Maekawa, Kenji Takenaka, Hitoshi Ichimiya, Reiji Terasaka

**Affiliations:** 1 Department of Preventive Medicine, Graduate School of Medical Sciences, Kyushu University, Fukuoka, Japan; 2 Department of Geriatric Medicine, Graduate School of Medical Sciences, Kyushu University, Fukuoka, Japan; 3 Department of Preventive Medicine, Nagoya University Graduate School of Medicine, Nagoya, Japan; 4 Department of Epidemiology and International Health, International Clinical Research Center, National Center for Global Health and Medicine, Tokyo, Japan; 5 Surgery and Oncology, Graduate School of Medical Sciences, Kyushu University, Fukuoka, Japan; 6 Surgery and Science, Graduate School of Medical Sciences, Kyushu University, Fukuoka, Japan; 7 Department of Gastroenterological Surgery, National Kyushu Cancer Center, Fukuoka, Japan; 8 Division of Surgery, National Kyushu Medical Center, Fukuoka, Japan; 9 Department of Surgery, Fukuoka University Chikushi Hospital, Chikushino, Japan; 10 Department of Regenerative Medicine & Transplantation Faculty of Medicine, Fukuoka University School of Medicine, Fukuoka, Japan; 11 Second Department of Surgery, Fukuoka University School of Medicine, Fukuoka, Japan; 12 Division of Surgery, Fukuoka City Hospital, Fukuoka, Japan; 13 Division of Surgery, Hamanomachi General Hospital, Fukuoka, Japan; 14 Division of Surgery, Fukuoka Red Cross Hospital, Fukuoka, Japan

**Keywords:** *XRCC1* polymorphisms, alcohol consumption, colorectal cancer

## Abstract

**Background:**

X-ray cross-complementing group 1 (*XRCC1*) polymorphisms affect DNA repair capacity and may therefore be of importance in colorectal carcinogenesis. Alcohol consumption, an important risk factor for colorectal cancer, may induce carcinogenesis through DNA damage caused by the toxic effects of alcohol or its metabolites. Therefore, we examined the associations of *XRCC1* Arg399Gln, Arg280His, and Arg194Trp polymorphisms with colorectal cancer and the impact of the association between alcohol consumption and colorectal cancer risk.

**Methods:**

This case-control study in Fukuoka, Japan including 685 cases and 778 controls. The cases were incident patients with histologically confirmed colorectal adenocarcinoma. The controls were randomly selected community subjects.

**Results:**

The *XRCC1* 399Gln/Gln genotype was significantly associated with colorectal cancer risk (adjusted odds ratio [OR] 1.57, 95% CI 1.01–2.42; relative to 399Arg/Arg genotype). The association was strongest in individuals with high alcohol consumption. The Arg280His polymorphism modified the association between alcohol consumption and colorectal cancer risk (interaction *P* = 0.049). The OR of colorectal cancer in individuals with the 280His allele was 0.45 (95% CI 0.26–0.78) as compared with the 280Arg/Arg genotype limited to the 399Gln allele (interaction *P* = 0.001). The adjusted ORs for 399Gln/Gln-280Arg/Arg-194Arg/Arg and 399Arg/Gln-280Arg/Arg-194Arg/Trp were 1.71 (95% CI 1.02–2.87) and 1.57 (95% CI 1.05–2.33), respectively, with 399Arg/Arg-280Arg/Arg-194Arg/Arg as reference (interaction *P* = 0.418).

**Conclusions:**

The findings are additional evidence that individuals with the *XRCC1* 399Gln/Gln genotype have an increased risk of colorectal cancer, and that *XRCC1* polymorphisms have an important role in colorectal cancer risk associated with alcohol consumption or gene-gene interaction.

## INTRODUCTION

Colorectal cancer is one of the most common cancers in North America and Western Europe,^1^^,^^2^ and its incidence has been increasing in Japan.^2^ The human X-ray repair cross-complementing group 1 (*XRCC1*) gene is a DNA base-excision repair gene that has been mapped to human chromosome 19q13.^3^ Human XRCC1 exhibits a number of isoenzymes, 3 of which result from known amino acid substitutions in the *XRCC1* gene, namely Arg194Trp (rs1799782), Arg280His (rs25489), and Arg399Gln (rs25487).^4^ The *XRCC1* 399Gln allele has been shown to be associated with measurably reduced DNA repair capacity, as assessed by persistence of DNA adducts, increased GPA somatic mutations,^5^ and elevated *p*53 mutations.^6^ However, the function of the Arg194Trp and Arg280His polymorphisms remains unclear. The base-excision repair pathway is designed to remove non-bulky base adducts produced by methylation, oxidation, and reduction by ionizing radiation or oxidative damage.^7^^,^^8^

Findings regarding the associations of these polymorphisms with colorectal cancer risk have been inconsistent. For the *XRCC1* 399Gln allele, 2 previous studies showed an increased risk of colorectal cancer,^9^^,^^10^ while other case-control studies showed no positive association with the that allele,^11^^–^^17^ including a small study in Japan.^13^ Discrepant findings have also been reported for the *XRCC1* Arg194Trp and Arg280His polymorphisms. Regarding Arg194Trp, a recent case-control study found that it was associated with a modest increase in the risk of colon cancer,^11^ but this association was not observed in other studies.^9^^,^^12^^,^^14^^–^^16^ Only 1^15^ of several studies^11^^,^^14^^,^^15^^,^^18^ showed an association between the Arg280His polymorphism and colorectal carcinogenesis.

Alcohol consumption is a known risk factor of colorectal cancer.^19^^,^^20^ However, our previous study^21^ showed that the *ADH1B* and *ALDH2* polymorphisms did not modify the association between alcohol consumption and colorectal cancer risk. Alcohol intake is associated with the production of reactive oxygen species—including oxygen radicals, lipid peroxidation, and acetaldehyde—which cause DNA damage that can be repaired by the DNA base-excision repair pathway.^22^ Therefore, polymorphisms of the base-excision repair gene (*XRCC1*) may be a risk factor for colorectal cancer and modify the association between alcohol consumption and colorectal cancer risk.

In the present study, we examined the associations of these 3 genetic polymorphisms of the *XRCC1* gene with colorectal cancer and the impact of the association between alcohol consumption and colorectal cancer risk in Japan.

## METHODS

The Fukuoka Colorectal Cancer Study was a case-control study of incident colorectal cancer patients and community control subjects residing in Fukuoka City and 3 adjacent areas. The details of the study were reported in our previous article,^23^ and the methods relevant to the present analysis are described below. The study protocol was approved by the ethics committees of Kyushu University and all participating hospitals, except for 2 that did not have ethics committees. The surveys at these hospitals were conducted with the permission of the respective hospital directors.

### Subjects

The cases comprised a consecutive series of patients with histologically confirmed incident colorectal adenocarcinoma who were admitted to 1 of 8 centers (2 university hospitals and 6 affiliated hospitals) for surgical treatment between October 2000 and December 2003. Other eligibility criteria included age 20 to 74 years at the time of diagnosis; residence in the study area; and no history of partial or total removal of the colorectum, familial adenomatous polyposis, or inflammatory bowel disease. Research nurses visited each hospital weekly and determined the eligibility of cases by referring to admission logs and medical records. Research nurses contacted each eligible patient with permission from an attending doctor and interviewed the patient after obtaining written informed consent. Of 1053 eligible patients, 840 (80%) participated in the interview, and 685 (65%) gave informed consent to genotyping. Reasons for nonparticipation were patient refusal (*n* = 115), refusal by the patient's physician (*n* = 46), and failure to make contact (*n* = 52).

Eligibility criteria for control subjects were the same as those for case patients, except for diagnosis of colorectal cancer and age 20 to 74 years at the time of selection. A total of 1500 persons were selected as control candidates by 2-stage random sampling from among residents living in 15 communities. Of these, individuals meeting any of the following criteria were excluded: death (*n* = 7), migration from the study area (*n* = 22), undelivered mail (*n* = 44), mental incompetence (*n* = 19), history of partial or total removal of the colorectum (*n* = 21), diagnosis of colorectal cancer after the survey (*n* = 5), no response (*n* = 158), and refusal to participate (*n* = 391). Exclusion of the first 6 categories of outcome (*n* = 118) left 1382 eligible persons, of whom 833 (60%) participated in the interview and 778 (56%) gave informed consent to genotyping.

### Interview

Research nurses used a uniform questionnaire interviewed all subjects in person regarding lifestyle factors, including alcohol consumption, smoking, and physical activity. Interviews for case subjects were carried out in the hospital during admission, while those for controls were conducted mostly at public community halls or collaborating clinics. The referent time point was the date of the onset of symptoms or screening (for cases) or the time of the interview (for controls).

Habitual alcohol consumption 5 years before the referent time point was ascertained. Individuals reported the average number of days per week that alcohol was consumed and the average amount of alcohol per day of drinking. Alcohol consumption was measured in conventional units: 1 *go* (180 ml) of *sake*, 1 large bottle (633 ml) of beer, half a *go* (90 ml) of *shochu*, 2 shots (60 ml in total) of whisky or brandy, and 2 glasses (200 ml in total) of wine were each expressed as 1 unit.

Detailed information on smoking history was elicited from individuals who had smoked cigarettes daily for 1 year or longer. Smoking history included the age at which the subject started and quit smoking, along with the number years of smoking and average number of cigarettes smoked per day for each decade of age from the second to the eighth decade. Cumulative exposure to cigarette smoking per decade was expressed as the product of the number of cigarettes smoked per day and the number of years of smoking in each decade.

Height (cm), current body weight (kg), and body weight 10 years before the study were reported. Body mass index (BMI, kg/m^2^) 10 years before the study was used in the analysis because current BMI is unrelated to colorectal cancer risk.^24^ Questions on physical activity elicited information on the type of job (sedentary or standing work; work involving walking, laboring, and hard laboring; and unemployment), activities in commuting and housework, and leisure-time activities 5 years before the study. Leisure-time physical activity was expressed as the product of metabolic equivalents (METs) and hours of weekly participation in each activity. Parental colorectal cancer was also elicited.

### Genotyping

A 5-ml venous blood sample was taken after the interview. DNA was extracted from the buffy coat using a commercial kit (Qiagen GmbH, Hilden, Germany), and genotyping was performed by a polymerase chain reaction-restriction fragment length polymorphism (PCR-RFLP) method. PCR was performed in a reaction mixture of 10 µl containing 0.5 units of Taq and 1 µl of template DNA with a concentration of 50 to 150 ng/µl. The *XRCC1* Arg194Trp and Arg399Gln genotypes were determined according to the methods described by Lunn et al.^5^ Primers for the Arg194Trp (rs1799782) gene were 5′-GCC CCG TCC CAG GTA-3′ (sense) and 5′-AGC CCC AAG ACC CTT TCA CT-3′ (antisense), and primers for the Arg399Gln (rs25487) gene were 5′-TTG TGC TTT CTC TGT GTC CA-3′ (sense) and 5′-TCC TCC AGC CTT TTC TGA TA-3′ (antisense). Both the 194Arg and 399Arg alleles create *Msp*I sites. PCR products were digested with *Msp*I (10 units) for 3 hours at 37°C in a mixture of 20 µl, resulting in fragments of 292 bp and 21 bp for the 194Arg allele and 313 bp for the 194Trp allele; and 374 bp and 221 bp for the 399Arg allele and 615 bp for the 399Gln allele. Arg280His (rs25489) was determined according to the method of Yin et al.^25^ 5′-CCC CAG TGG TGC TAA CCT AA-3′ (sense) and 5′-CTA CAT GAG GTG CGT GCT GT-3′ (antisense) primers were used. Twenty microliters of PCR product was digested with 10 units of *Rsa*I for 3 hours at 37°C, resulting in fragments of 246 bp and 58 bp for the 280Arg allele and 304 bp for the 280His allele. The digested PCR products were separated by electrophoresis on 3% agarose gels (NuSieve GTG, BMA, Rockland, ME, USA) and visualized with ethidium bromide.

### Statistical analysis

All statistical analyses were performed using SAS version 9.1 (SAS Institute Inc., Cary, NC, USA). Associations between the genetic polymorphisms and the risk of colorectal cancer were examined by multiple logistic regression analyses, which included indicator variables for sex, 10-year age class (lowest class <40 years), area of residence (Fukuoka City or adjacent areas), alcohol intake (0, 0.1–1.9, and ≥2 units/day), cigarette-years (never, <800, and ≥800), BMI 10 years before the study (<25 and ≥25 kg/m^2^), type of job (sedentary, moderate, and hard work), leisure-time physical activity (0, 0.1–15.9, and ≥16 MET-hours/week), and history of parental colorectal cancer as covariates.

Adjusted odds ratios (ORs) and 95% confidence interval (CIs) were obtained from the logistic regression coefficient and the standard error for the corresponding indicator variable. Statistical significance for the interaction was tested by the likelihood ratio test, which compared logistic models with and without interaction terms. Statistical significance was defined as a 2-sided *P*-value of less than 0.05.

Deviation from the Hardy-Weinberg equilibrium was evaluated by the chi-square test with 1 degree of freedom. The linkage disequilibrium was evaluated using the expectation-maximization algorithm. The OR for a specific combined genotype was obtained using logistic regression analysis with each combined genotype as an independent variable.

## RESULTS

Selected characteristics of the study subjects are summarized in Table 1. Case subjects were older than the controls and had higher prevalences of high BMI (≥25 kg/m^2^) 10 years before the study, heavy alcohol intake (≥2 units/day), and parental history of colorectal cancer. In contrast, sex, area of residence, cigarette-years, type of job, and leisure-time physical activity did not substantially differ between case and control subjects.

**Table 1.** Characteristics of case and control subjects

Characteristic	Cases (*n* = 685)	Controls (*n* = 778)	*P* value^a^
Sex, *n* (%)			0.755
Male	426 (62.2)	490 (63.0)	
Female	259 (37.8)	288 (37.0)	
Age (mean ± SD)	60.2 ± 9.1	58.6 ± 10.7	0.003
Age (years), *n* (%)			0.046
<40	16 (2.4)	40 (5.1)	
40–49	70 (10.2)	94 (12.1)	
50–59	218 (31.8)	233 (30.0)	
60–69	257 (37.5)	282 (36.2)	
70+	124 (18.1)	129 (16.6)	
Area, *n* (%)			0.223
Fukuoka	420 (61.3)	501 (64.4)	
Adjacent area	265 (38.7)	277 (35.6)	
BMI (kg/m^2^) 10 years previously, *n* (%)			0.030
<25	487 (71.1)	592 (76.1)	
≥25	198 (28.9)	186 (23.9)	
Alcohol consumption, *n* (%)^b^			0.036
Never	272 (39.7)	311 (40.0)	
<2 units/day	221 (32.3)	290 (37.3)	
≥2 units/day	192 (28.0)	177 (22.7)	
Cigarette-years, *n* (%)			0.362
Never	289 (42.2)	308 (39.6)	
<800	231 (33.7)	290 (37.3)	
≥800	165 (24.1)	180 (23.1)	
Type of job, *n* (%)^c^			0.503
Sedentary	495 (72.3)	542 (69.7)	
Moderate	87 (12.7)	113 (14.5)	
Hard	103 (15.0)	123 (15.8)	
Leisure-time physical activity, *n* (%)^c^			0.393
0.0	223 (32.6)	230 (29.6)	
0.1–15.9	237 (34.6)	271 (34.8)	
16+	225 (32.8)	277 (35.6)	
Parental colorectal cancer, *n* (%)	54 (7.9)	42 (5.4)	0.056

^a^Based on the chi-square test for proportion and analysis of variance for means.

^b^1 unit of alcohol intake corresponds to 1 *go* (180 ml) of *sake*, 0.5 *go* (90 ml) of *shochu*, 1 large bottle (633 ml) of beer, 2 shots (60 ml in total) of whiskey, or 2 glasses (200 ml in total) of wine.

^c^MET-hours/week.

The *XRCC1* Arg194Trp, Arg280His, and Arg399Gln genotypes were not determined in 2 controls, 1 case, and 2 controls, respectively. Genotype distributions of the Arg194Trp, Arg280His, and Arg399Gln polymorphisms in control subjects were in agreement with the Hardy-Weinberg equilibrium (*P* = 0.509 for Arg194Trp, *P* = 0.180 for Arg280His, and *P* = 0.245 for Arg399Gln). The 399Gln/Gln genotype was more frequent in cases than controls. The crude and adjusted ORs of colorectal cancer for the 399Gln/Gln genotype as compared with the 399Arg/Arg genotype were significantly higher than unity (Table 2). Regarding the Arg280His polymorphism, the 280His/His genotype was more frequent in cases than controls, although the overall number of subjects with this genotype was very low. The distribution of Arg194Trp genotypes between cases and controls did not significantly differ.

**Table 2.** Odds ratio (OR) and 95% confidence interval (95% CI) of *XRCC1* polymorphisms for colorectal cancer risk

Genotype	Cases (%)	Controls (%)	Crude OR (95% CI)	Adjusted OR^a^ (95% CI)
Arg194Trp^b^				
Arg/Arg	321 (46.9)	368 (47.4)	1 (reference)	1 (reference)
Arg/Trp	298 (43.5)	327 (42.2)	1.05 (0.84–1.30)	1.05 (0.84–1.30)
Trp/Trp	66 (9.6)	81 (10.4)	0.93 (0.65–1.34)	0.89 (0.62–1.28)
Arg280His^c^				
Arg/Arg	573 (83.8)	641 (82.4)	1 (reference)	1 (reference)
Arg/His	103 (15.0)	134 (17.2)	0.86 (0.65–1.14)	0.88 (0.66–1.17)
His/His	8 (1.2)	3 (0.4)	2.98 (0.79–11.28)	3.07 (0.80–11.79)
Arg399Gln^d^				
Arg/Arg	356 (52.0)	436 (56.2)	1 (reference)	1 (reference)
Arg/Gln	275 (40.1)	299 (38.5)	1.13 (0.91–1.40)	1.13 (0.91–1.41)
Gln/Gln	54 (7.9)	41 (5.3)	1.61 (1.05–2.48)	1.57 (1.01–2.42)

^a^Adjusted for sex, age, residence area, body mass index, alcohol consumption, cigarette smoking, type of job, leisure-time physical activity, and parental colorectal cancer.

^b^2 controls were excluded due to undetermined genotype.

^c^1 case was excluded due to undetermined genotype.

^d^2 controls were excluded due to undetermined genotype.

Table 3 summarizes the combined effects of the *XRCC1* polymorphisms and alcohol consumption on colorectal cancer risk. Because few individuals had the 280His/His genotype, individuals heterozygous for Arg280His were combined with those homozygous for the minor allele. An increased OR of colorectal cancer associated with the 399Gln/Gln genotype was most evident in those with high alcohol consumption (≥2 units/day); however, the gene-environment interaction was not statistically significant (interaction *P* = 0.614). Regarding the Arg280His polymorphism, a positive association between alcohol consumption and colorectal cancer was observed with the 280Arg/Arg genotype, but an inverse association was seen with the 280His alleles. The gene-environment interaction was statistically significant (interaction *P* = 0.049). Individuals with high alcohol consumption who had the 194Arg/Arg or Arg/Trp genotype had an increased risk as compared with those with no alcohol consumption who had the 194Arg/Arg genotype; however, the gene-environment interaction was not statistically significant (interaction *P* = 0.503).

**Table 3.** Combined effect of alcohol consumption and *XRCC1* polymorphisms on colorectal cancer risk

*XRCC1*		Alcohol intake (units/day)			Interaction

		Never	<2	≥2	
Arg194Trp					*P* = 0.503
Arg/Arg	No.^a^	126/152	106/135	89/81	
	OR (95% CI)^b^	1.00 (reference)	1.07 (0.74–1.55)	1.52 (1.00–2.31)	
Arg/Trp	No.^a^	123/130	92/126	83/71	
	OR (95% CI)^b^	1.16 (0.82–1.64)	0.99 (0.68–1.44)	1.64 (1.06–2.53)	
Trp/Trp	No.^a^	23/29	23/27	20/25	
	OR (95% CI)^b^	0.94 (0.51–1.72)	1.13 (0.60–2.10)	1.07 (0.55–2.08)	
Arg280His					*P* = 0.049
Arg/Arg	No.^a^	225/267	181/232	167/142	
	OR (95% CI)^b^	1.00 (reference)	1.02 (0.77–1.35)	1.57 (1.13–2.19)	
Arg/His + His/His	No.^a^	46/44	40/58	25/35	
	OR (95% CI)^b^	1.25 (0.79–1.97)	0.93 (0.59–1.46)	0.95 (0.54–1.69)	
Arg399Gln					*P* = 0.614
Arg/Arg	No.^a^	138/173	123/162	95/101	
	OR (95% CI)^b^	1.00 (reference)	1.05 (0.75–1.48)	1.34 (0.90–1.99)	
Arg/Gln	No.^a^	110/117	81/111	84/71	
	OR (95% CI)^b^	1.20 (0.85–1.70)	1.04 (0.71–1.52)	1.68 (1.10–2.56)	
Gln/Gln	No.^a^	24/21	17/15	13/5	
	OR (95% CI)^b^	1.36 (0.72–2.57)	1.52 (0.73–3.19)	3.70 (1.26–10.84)	

^a^Numbers of cases/controls.

^b^Adjusted for sex, age, residence area, body mass index, cigarette smoking, type of job, leisure-time physical activity, and parental colorectal cancer.

The effects of the Arg399Gln polymorphism according to the Arg194Trp or Arg280His polymorphism, and the association between Arg194Trp and Arg280His were examined (Table 4). Individuals heterozygous with the corresponding polymorphisms were combined with those homozygous for each minor allele, because greater statistical power is necessary to investigate gene-gene interaction. The 280His allele was associated with an OR for colorectal cancer of 0.45 (95% CI 0.26–0.78) as compared with the 280Arg/Arg genotype limited to the 399Gln allele. Gene-gene interaction was statistically significant (interaction *P* = 0.001). In contrast, no clear interaction with regard to risk was seen between the Arg194Trp and either the Arg399Gln or Arg280His polymorphism (*P* for interaction, 0.254 and 0.880, respectively).

**Table 4.** Effect of *XRCC1* Arg194Trp or Arg280His polymorphism by *XRCC1* Arg399Gln polymorphism on the risk of colorectal cancer, and the association between Arg194Trp and Arg280His polymorphisms for the risk

Genotype		Arg399Gln		Interaction

		Arg/Arg	Arg/Gln + Gln/Gln	
Arg194Trp				*P* = 0.254
Arg/Arg	No.^a^	232/290	132/118	
	OR (95% CI)^b^	1 (reference)	1 (reference)	
Arg/Trp + Trp/Trp	No.^a^	124/146	197/222	
	OR (95% CI)^b^	1.07 (0.79–1.45)	0.83 (0.60–1.14)	
Arg280His				*P* = 0.001
Arg/Arg	No.^a^	267/348	306/293	
	OR (95% CI)^b^	1 (reference)	1 (reference)	
Arg/His + His/His	No.^a^	89/88	22/47	
	OR (95% CI)^b^	1.33 (0.95–1.87)	0.45 (0.26–0.78)	

		Arg280His		
		
		Arg/Arg	Arg/His + His/His	

Arg194Trp				*P* = 0.880
Arg/Arg	No.^a^	323/362	41/46	
	OR (95% CI)^b^	1 (reference)	1 (reference)	
Arg/Trp + Trp/Trp	No.^a^	250/279	70/89	
	OR (95% CI)^b^	1.00 (0.79–1.25)	1.08 (0.63–1.87)	

^a^Numbers of cases/controls.

^b^Adjusted for sex, age, residence area, body mass index, alcohol intake, cigarette smoking, type of job, leisure-time physical activity, and parental colorectal cancer.

Lewontin’s *D*′ was 0.91, 0.94, and 0.93 for the linkage disequilibrium between Arg399Gln and Arg280His, Arg399Gln and Arg194Trp, and Arg280His and Arg194Trp, respectively. The estimated frequencies of the combined genotypes in cases and controls are shown in Table 5. The adjusted ORs for 399Gln/Gln-280Arg/Arg-194Arg/Arg and 399Arg/Gln-280Arg/Arg-194Arg/Trp were 1.71 (95% CI 1.02–2.87) and 1.57 (95% CI 1.05–2.33), respectively, with 399Arg/Arg-280Arg/Arg-194Arg/Arg as the reference. However, the interaction was not statistically significant (*P* = 0.418).

**Table 5.** Odds ratio (OR) and 95% confidence interval (95% CI) of colorectal cancer based on combination of *XRCC1* genotypes

*XRCC1* Combined genotypes^a^	Cases (%)	Controls (%)	Crude OR (95% CI)	Adjusted OR^b^ (95% CI)
399Arg/Arg-280Arg/Arg-194Arg/Arg	75 (10.9)	102 (13.1)	1 (reference)	1 (reference)
399Arg/Arg-280Arg/His-194Arg/Arg	41 (6.0)	41 (5.3)	1.36 (0.80–2.30)	1.49 (0.87–2.53)
399Arg/Arg-280His/His-194Arg/Arg	8 (1.2)	3 (0.4)	3.63 (0.93–14.13)	3.72 (0.94–14.7)
399Arg/Gln-280Arg/Arg-194Arg/Arg	124 (18.1)	136 (17.5)	1.24 (0.84–1.82)	1.30 (0.88–1.93)
399Arg/Gln-280Arg/His-194Arg/Arg	19 (2.8)	45 (5.8)	0.57 (0.31–1.06)	0.62 (0.33–1.16)
399Gln/Gln-280Arg/Arg-194Arg/Arg	51 (7.5)	41 (5.3)	1.69 (1.02–2.81)	1.71 (1.02–2.87)
399Gln/Gln-280Arg/His-194Arg/Arg	2 (0.3)	0	—	—
399Arg/Arg-280Arg/Arg-194Arg/Trp	130 (19.0)	170 (21.9)	1.04 (0.71–1.51)	1.12 (0.76–1.64)
399Arg/Arg-280Arg/His-194Arg/Trp	39 (5.7)	43 (5.5)	1.23 (0.73–2.09)	1.23 (0.72–2.11)
399Arg/Gln-280Arg/Arg-194Arg/Trp	127 (18.6)	112 (14.4)	1.54 (1.04–2.28)	1.57 (1.05–2.33)
399Arg/Gln-280Arg/His-194Arg/Trp	1 (0.1)	2 (0.3)	0.68 (0.06–7.64)	0.55 (0.05–6.37)
399Gln/Gln-280Arg/Arg-194Arg/Trp	1 (0.1)	0	—	—
399Arg/Arg-280Arg/Arg-194Trp/Trp	62 (9.1)	76 (9.9)	1.11 (0.71–1.74)	1.09 (0.69–1.72)
399Arg/Arg-280Arg/His-194Trp/Trp	1 (0.2)	1 (0.1)	1.36 (0.08–22.1)	2.28 (0.13–38.59)
399Arg/Gln-280Arg/Arg-194Trp/Trp	3 (0.4)	4 (0.5)	1.02 (0.22–4.69)	1.12 (0.23–5.39)

^a^The frequencies of 399Arg/Gln-280His/His-194Arg/Arg, 399Gln/Gln-280His/His-194Arg/Arg, 399Arg/Arg-280His/His-194Arg/Trp, 399Arg/Gln-280His/His-194Arg/Trp, 399Gln/Gln-280Arg/His-194Arg/Trp, 399Gln/Gln-280His/His-194Arg/Trp, 399Arg/Arg-280His/His-194Trp/Trp, 399Arg/Gln-280Arg/His-194Trp/Trp, 399Arg/Gln-280His/His-194Trp/Trp, 399Gln/Gln-280Arg/Arg-194Trp/Trp, 399Gln/Gln-280Arg/His-194Trp/Trp, and 399Gln/Gln-280His/His-194Trp/Trp were zero in both cases and controls.

^b^Adjusted for sex, age, residence area, body mass index, alcohol intake, cigarette smoking, type of job, leisure-time physical activity, and parental colorectal cancer.

## DISCUSSION

In this study, we investigated the associations between genetic polymorphisms in the DNA repair gene *XRCC1* and colorectal cancer risk. The results showed that the *XRCC1* 399Gln/Gln genotype was associated with an increased risk of colorectal cancer and that the increase was greatest in those with high alcohol consumption. Alcohol consumption was positively associated with risk in individuals with the *XRCC1*280Arg/Arg genotype, but inversely associated with risk in those with *XRCC1* 280His alleles. We also found that individuals with the 399Gln allele and 280His allele had a lower risk of colorectal cancer. In addition, we observed an increased colorectal cancer risk in individuals with the combined genotypes of 399Gln/Gln-280Arg/Arg-194Arg/Arg and 399Arg/Gln-280Arg/Arg-194Arg/Trp, as compared with individuals with 399Arg/Arg-280Arg/Arg-194Arg/Arg.

A study in Egypt reported that individuals with the *XRCC1* 399Gln allele had an approximately 4-fold risk of colorectal cancer as compared with those with the *XRCC1* 399Arg/Arg genotype,^9^ although the number of subjects was small. In a case-control study in South Korea, individuals with the *XRCC1* 399Gln allele had a 2-fold higher OR of colorectal cancer than those with the *XRCC1* 399Arg/Arg genotype.^10^ Our present findings are consistent with those 2 previous studies. In contrast, other case-control studies in various countries^11^^–^^17^ did not observe a positive association between the *XRCC1* 399Gln allele and the risk of colorectal cancer.

Several studies have examined the association between the *XRCC1* Arg280His polymorphism and colorectal cancer risk and found no measurable association.^11^^,^^14^^,^^15^^,^^18^ In the present study, the Arg280His polymorphism was not significantly associated with the overall risk of colorectal cancer, although a modifying effect between risk and alcohol consumption was observed. Furthermore, we found a gene-gene interaction between the Arg280His and Arg399Gln polymorphisms and risk. We therefore infer that these polymorphisms are associated with alcohol consumption-mediated colorectal carcinogenesis. Individuals with the 280His/His genotype had approximately 3-fold the risk of colorectal cancer as those with 280Arg/Arg genotype (Table 2). Nevertheless, given the small number of individuals with the 280His/His genotype, this finding might simply be within the range of normal variation.

With regard to the *XRCC1* 194Trp allele, a large case-control study in the United States reported that this allele was associated with a modest increase in the risk of colon cancer,^11^ while another study showed that it had an insignificant modifying effect on the association between alcohol consumption and risk.^16^ However, most studies found that the 194Trp allele was not associated with an increased risk of colorectal cancer,^9^^,^^12^^,^^14^^–^^16^ and the present study observed no association between the Arg194Trp polymorphism and risk. Individuals with the 194Arg allele showed a moderately increased risk of colorectal cancer with high alcohol consumption, but this may have been solely due to the effect of high alcohol consumption on colorectal carcinogenesis.

The inconsistency of the findings from studies of the association between the 3 polymorphisms and colorectal cancer risk may be due to the linkage with functional genotypes of *XRCC1*. A study reported linkage between Arg399Gln and Arg194Trp, by showing that carrying the 399Arg allele appeared on the 194Trp allele in Taiwanese.^6^ We also observed a close linkage among the 3 polymorphisms. The 194Trp allele was found to be linked to the 399Arg allele and 280Arg allele, while the 280His allele was strongly linked to the 399Arg allele. Conversely, the 399Gln allele was linked to 280Arg and 194Arg. Therefore, the risk of colorectal cancer might be attributable to the combined genotypes of *XRCC1*.

An interaction effect between *XRCC1* polymorphisms and alcohol consumption on colorectal cancer risk is biologically plausible. Alcohol intake is associated with the production of reactive oxygen species, including oxygen free radicals, which may generate DNA base lesions.^26^^,^^27^ Alcohol is converted to acetaldehyde in the colonic lumen, which induces the formation of DNA adducts and produces oxidative DNA damage.^28^ Human mammary epithelial cells exposed to ethanol showed a decreased capacity to remove benzo[a]pyrene diol-epoxide (BPDE)-DNA adducts,^29^ and ethanol and acetaldehyde impair the repair of bleomycin-damaged DNA in humans.^30^ Furthermore, alcohol and acetaldehyde adversely affect the metabolism of folate,^31^ which has been suggested to decrease the risk of colorectal cancer and adenoma.^32^^,^^33^ Depletion of 5,10-methylenetetrahydrofolate results in uracil misincorporation into DNA, and removal of this abnormal base may lead to single- and double-strand breaks.^34^

Cigarette smoking is another important environmental risk factor in colorectal cancer.^35^ In this dataset, there was no association between cigarette smoking and colorectal cancer risk.^36^ In addition, we found no interaction between cigarette smoking and *XRCC1* polymorphisms in the risk of colorectal cancer (data not shown).

Several methodological strengths of the present study warrant mention. First, this is the largest published study to examine the association between *XRCC1* polymorphisms and colorectal cancer in Japan. Among previous large studies, 1 study in the United States included 1604 patients with colon cancer and 1969 control subjects.^11^ Another in Taiwan investigated 727 case and 736 controls.^17^ Sample size is particularly important in investigating the role of rare genotypes in gene-environment or gene-gene interactions. Second, our study used community controls and an ethnically homogeneous population. Third, although we used alcohol consumption 5 years before the referent date, recall of this information was found to be highly reproducible and valid.^37^

The methodological weaknesses of the study were as follows. First, participation in genotyping was not particularly high for either cases (65%) or controls (56%). However, the frequency of the *XRCC1* 399Gln allele (25%) was similar to that reported in other Japanese populations,^13^^,^^38^ and the frequency of the *XRCC1* 194Trp allele (32%) was consistent with the results of a study in Japanese (30%).^39^ Information on the frequency of the *XRCC1* 280His allele in a Japanese population was not available because, to our knowledge, the present study is the first to report an association between the *XRCC1* Arg280His polymorphism and cancer risk in Japan. However, the frequency of the *XRCC1* 280His allele (9%) in our study was similar to that in Asian/Pacific islanders (9%).^40^ Second, because the community controls were not strictly investigated for the absence of colorectal cancer, such as by colonoscopy, we cannot exclude the possibility of misclassification of disease status. In addition, there are other DNA repair pathways (eg, base-excision repair, nucleotide-excision repair, mismatch repair, homologous recombination, and non-homologous end-joining), which are associated with many genetic polymorphisms, such as *OGG1*, *XPD*, *XPC*, *MSH6*, *XRCC3*, and *XRCC4*. However, we analyzed only *XRCC1* polymorphisms in this study. It is necessary to examine associations between other polymorphisms of DNA repair gene and colorectal cancer risk in the future.

In conclusion, the findings add evidence to the hypothesis that individuals with the *XRCC1* 399Gln/Gln genotype are at increased risk of colorectal cancer and that *XRCC1* polymorphisms have an important role in colorectal cancer risk related to alcohol consumption or gene-gene interaction.
